# Impact of external cooling with icepacks on ^68^Ga-PSMA uptake in salivary glands

**DOI:** 10.1186/s13550-018-0408-2

**Published:** 2018-07-03

**Authors:** Ludwike W. M. van Kalmthout, Marnix G. E. H. Lam, Bart de Keizer, Gerard C. Krijger, Tessa F. T. Ververs, Rememrt de Roos, Arthur J. A. T. Braat

**Affiliations:** 1 Department of Radiology and Nuclear Medicine, University Medical Center Utrecht, Utrecht, Netherlands; 2 Department of Clinical Pharmacy, University Medical Center Utrecht, Utrecht, Netherlands

## Abstract

**Background:**

External cooling of the salivary glands is advised to prevent xerostomia in lutetium-177-PSMA treatment for advanced prostate cancer. Since evidence addressing this subject is sparse, this study aims to determine impact of icepacks application on uptake in salivary glands. Eighty-nine patients referred for gallium-68-PSMA PET/CT for (re)staging of prostate cancer were prospectively included. Twenty-four patients were scanned with unilateral (solely left-sided) icepacks; 20 with bilateral icepacks; 45 without icepacks. Icepacks were applied approximately 30 minutes prior to tracer injection. PET/CT acquisition started 1 hour postinjection. Radiotracer uptake was measured in the parotid- and submandibular glands.

**Results:**

When comparing the intervention group with the control group, uptake in the left parotid gland significantly differed: SUV_max_: 11.07 ± 3.53 versus 12.95 ± 4.16; *p* = 0.02. SUV_peak_: 9.91 ± 3.14 versus 11.45 ± 3.61; *p* = 0.04. SUV_max_ and SUV_peak_ were reduced with 14.52% and 13.45%. All other SUV values did not significantly differ. Patients with bilateral icepacks showed no significant differences in PSMA uptake compared to the control group (all: *p* > 0.05). Intra-patient analysis revealed some significant differences in SUV_max_ and SUV_peak_ between the cooled and non-cooled parotid gland (SUV_max_: 11.12 ± 3.71 versus 12.69 ± 3.75; *p* = 0.00. SUV_peak_: 9.93 ± 3.32 versus 11.25 ± 3.25; *p* = 0.00).

**Conclusions:**

Impact of icepacks on PSMA uptake seems to be limited to the parotid glands. As clinical relevance of these findings is debatable, structural application of icepacks in the setting of lutetium-177 PSMA therapy needs careful consideration.

## Background

Prostate-specific membrane antigen (PSMA)-targeting radiotracers have gained popularity over the last years in the setting of prostate carcinoma diagnosis (Gallium-68 (^68^Ga) PSMA) and treatment (Lutetium-177 (^177^Lu) PSMA). PSMA is a transmembrane glycoprotein that is expressed by epithelial cells of the prostate. PSMA is 100–1000 times upregulated in prostate carcinoma cells, compared to benign prostate tissue. Its expression is directly correlated with the tumor’s aggressiveness [1]. Therefore, it represents an attractive target for diagnosis and treatment of prostate carcinoma using radioligands: so-called peptide receptor ligand therapy (PRLT). ^177^Lu-PSMA is a low molecular weight ligand that binds to the cell surface of prostate cancer cells. It is subsequently transported into the cell by receptor-mediated endocytosis, resulting in beta-emission and local radiation of prostate cancer cells to both the primary tumor and (distant) metastases.

Initially believed to be prostate specific, the PSMA receptor is expressed by other, both benign and malignant, tissues including the kidneys (proximal tubules), the jejunum (brush border), astrocytes, and Schwann cells in the central nervous system, ductal epithelium of breast tissue, and skeletal muscle [2]. In addition, significant PSMA expression is evident in the salivary glands: mean SUV_max_ in the parotid and submandibular glands 1 h post-injection was found to be 13.8 (9.0–28.3) and 14.5 (7.2–27.5), respectively [3]. Preliminary research in our institution also found a physiological high tracer accumulation in the salivary glands in 30 consecutive patients who underwent ^68^Ga-PSMA PET/CT for (re)staging of prostate cancer. Mean SUV_max_ in the parotid and submandibular glands 1 h post-injection was 12.3 (range 5.2–22.9) and 11.7 (range 6.0–22.2), respectively [4].

The high accumulation of therapeutic radioligands in the salivary glands may result in the frequently observed, undesirable side effect xerostomia. External cooling of the salivary glands is hypothesized to cause vasoconstriction, reduce blood flow, and decrease PSMA uptake in the salivary glands to ultimately prevent the salivary glands for radiation toxicity [5]. Therefore, external cooling of the salivary glands with icepacks, considered to be a harmless and well-tolerable procedure, is currently performed in clinical practice [6]. To date however, there has been no established evidence that cooling indeed decreases PSMA uptake in the salivary glands, without additional patient discomfort.

Since it is expected that ^177^Lu-PSMA therapy will be more widely applied over the next years, universal optimization of per-procedural scan protocols are needed. This study aims to clarify the impact of cooling with icepacks on PSMA uptake in salivary glands to guide ^177^Lu-PSMA treatment in the future.

## Methods

### Study population

Patients referred for a ^68^Ga-PSMA-11 PET/CT for (re)staging of prostate cancer were consecutively included in this analysis from September 2016 up to March 2017, after obtaining informed consent. We first included the intervention group: 20 patients who were scanned with bilateral icepacks, followed by 24 patients who were scanned with unilateral (solely left-sided) icepacks. The control group included 45 patients who were retrospectively included. Patients who underwent previous radiation therapy on the head/neck region were excluded from analysis.

### ^68^Ga-PSMA-11 preparation

^68^Ga-PSMA-11 was prepared using a GMP-grade ^68^Ge/^68^Ga generator and a semi-automated Modular-Lab eazy synthesis module (Eckert & Ziegler, Berlin, Germany). Each synthesis was performed following the manufacturers’ instructions using prefabricated materials including a cassette, an acetate buffer, a C18 purification cartridge, and a 0.22-μm pore size sterilization filter (Eckert & Ziegler, Berlin, Germany). Forty micrograms (42 nmol) of PSMA-11 ligand (ABX, Radeberg, Germany) per preparation was used, leading to the mean administered amount of 14.5 (range 5.1–41.1) μg ligand per patient depending on patient weight and ^68^Ge decay.

### PET/CT acquisition and image reconstruction

**Fig. 1:**
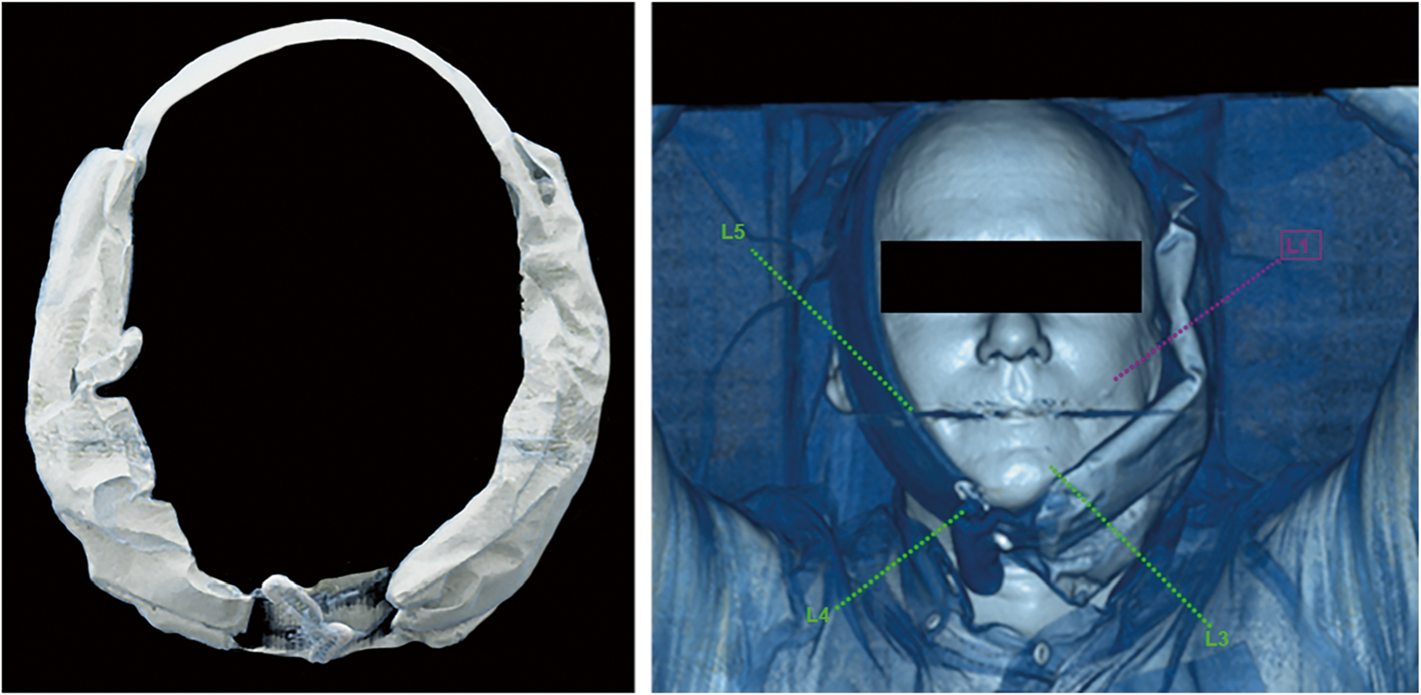
The cooling device as used in this study (left); a patient scanned with a unilateral (left-sided) icepack (right)

Images were acquired from skull vertex to the thighs using a Biograph mCT40 scanner (Siemens, Erlangen, Germany). After intravenous injection of 2 MBq/kg ^68^Ga-PSMA-11, 500 ml of saline was intravenously administered. Frozen icepacks were placed in an in-house made synthetic cover, allowing for full coverage of the targeted salivary glands and effective fixation to the patient’s face. An example of this cooling device is shown in Fig. 1. In the patient group scanned with unilateral icepacks, the icepack was exclusively placed on the left side of the cover. The icepacks were applied approximately 30 min prior to tracer injection up to termination of the scanning procedure (approximately 100 min). All icepacks were replaced for new ones 30 min prior to PET/CT acquisition to ensure continuous, effective cooling of the salivary glands.

PET images were acquired 45 min after radiotracer administration. A low dose CT was performed directly following PET imaging, approximately 60 min after radiotracer administration. Images were acquired according to the European Association of Nuclear Medicine (EANM) criteria, a.k.a. EARL-reconstructions, with the following parameters: PET with time-of-flight and point spread function reconstruction, 4 iterations, 21 subsets, with a filter of 7.5 mm full width at half maximum [7].

### Image analysis

**Fig. 2:**
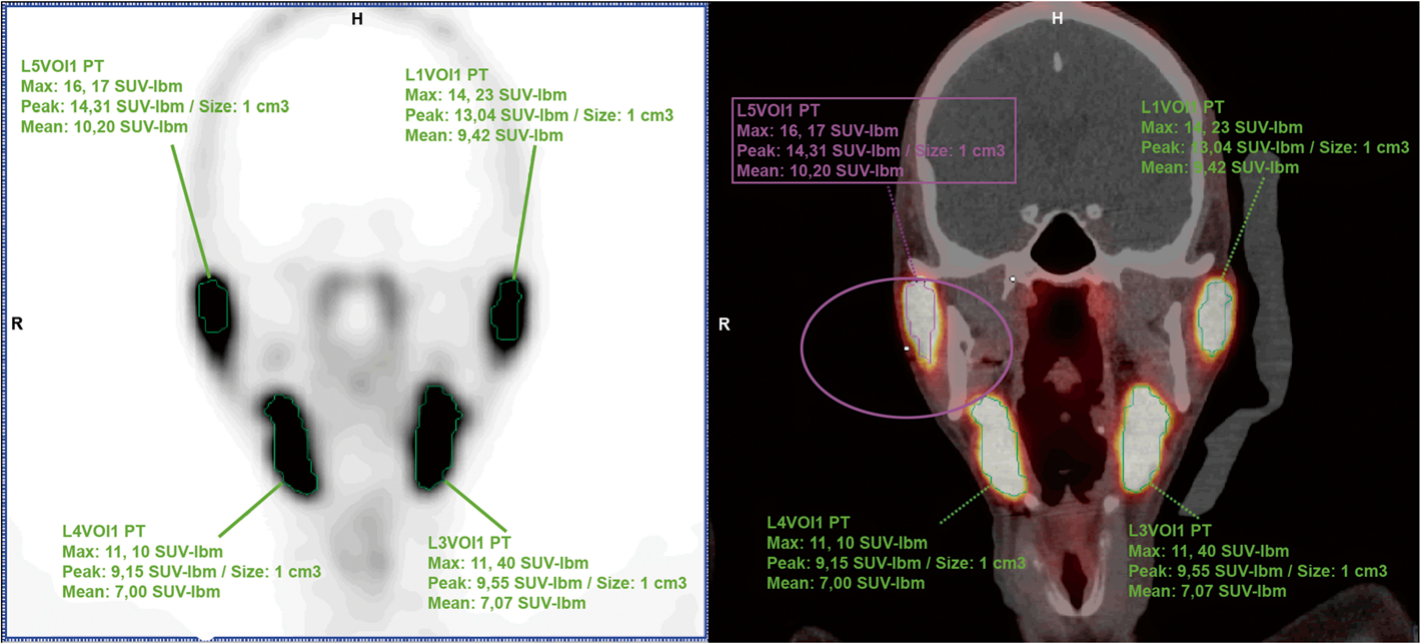
Measurements on PSMA uptake in both parotid glands in a patient who was scanned with a left-sided icepack. Differences in PSMA uptake were observed, when comparing the cooled (left) side to the non-cooled (right) side

Radiotracer uptake in the parotid glands and the submandibular glands was quantitatively assessed, using a commercial software package (Syngo.via; Siemens Healthcare). Mean, peak, and maximum standardized uptake values (SUV_max_, SUV_peak_, and SUV_mean_) were measured by placing a 3D volume of interest (VOI) within bilateral parotid and submandibular gland region on the PET/CT images (Fig. 2). Salivary glands were delineated using a 10% threshold of the maximum pixel value within the VOI (isocontour). Measurements were corrected for lean body mass, according to the formula as defined in the EANM guidelines [8]. Measurements were executed by one involved researcher and were randomly checked by an experienced nuclear medicine physician. Blinding of images, masking the applied icepacks, was not performed.

### Statistical analysis

Statistical analyses were performed using IBM SPSS Statistics for Mac, version 24. Aiming at a PSMA uptake reduction of 40% by the application of icepacks and assuming a type I error of 0.05, a power analysis revealed the required number of patients for comparison to be 45 in the intervention group and 45 in the control group. Means of continuous data at baseline were compared using the one-way ANOVA test. Means of nominal data were analyzed using the chi-square test. To compare mean radiotracer uptake in the bilateral icepack group with the control group, an independent *T* test was used. A paired *T* test was used to compare inpatient radiotracer uptake differences in the unilateral icepack group. Statistical significance was established for *p* values of < 0.05.

## Results

A total number of 71 patients was approached for inclusion. Twenty-five patients did not agree with inclusion; 2 patients were excluded because of a previous history of radiation therapy to the head/neck region. Twenty-four patients were scanned with a unilateral icepack on the left side, allowing intra-individual analysis, and 20 patients were scanned with bilateral icepacks, totaling 44 patients in the intervention group. A control group of 45 patients was scanned without icepacks, in line with regular clinical practice. Baseline characteristics of the included patient groups are presented in Table 1. Comparison of various baseline criteria did not reveal any statistical differences.

**Table 1:** Baseline characteristics

Subjects	Intervention group (*N* = 44)		Control group (*N* = 45)	
	Left-sided icepacks (*N* = 24)	Bilateral icepacks (*N* = 20)	No icepacks (*N* = 45)	*p* value*
Age (years)				
Mean (± SD)	72.79 (± 4.86)	71.00 (± 5.91)	70.67 (± 7.57)	0.46
Range (years)	64–81	62–82	49–91	
Initial Gleason Score (*N*)	24 (0 missing)	18 (2 missing)	41 (4 missing)	0.17
Low risk(< 7)	9	9	8	
Intermediate risk (7)	7	5	19	
High risk (> 7)	8	4	14	
PSA at scan (ng/ml)				
Mean (± SD)	24.3 (± 35.23)	11.79 (± 10.61)	18.17 (± 31.68)	0.38
Range	1.10–164.7	0.12–35.0	0.20–120.0	
Scan indication (*N*)	24 (0 missing)	20 (0 missing)	45 (0 missing)	
- Primary staging	5	2	4	
- Re-staging	19	17	35	
- Other indications	0	1	6	
Previous therapy regimens (*N*)	24 (0 missing)	19 (1 missing)	45 (0 missing)	
- No treatment	5	0	4	
- Radical prostatectomy	3	6	17	
- Radiation therapy	10	11	12	
- Radiation therapy + hormonal therapy (Bolla [23])	2	1	7	
- Other treatment regimens	4	1	5	
Adjuvant/salvage (radiation/hormonal) therapy regimens (*N*)	24 (0 missing)	17 (3 missing)	44 (1 missing)	0.34
- Yes	9	3	11	
- No	15	14	33	

*p < 0.05 is considered significant

### Radiotracer activity in the intervention group versus control group

When comparing SUV values in the intervention group (bilateral + unilateral icepack group) with the control (no icepacks) group, significant differences were found with regard to radiotracer uptake in the left parotid gland. In the control group, SUV_max_ in the left parotid gland was 12.95 ± 4.16, versus 11.07 ± 3.53 in the intervention group (*p* = 0.02). Absolute reduction was 14.52%. SUV_peak_ in the control group was 11.45 ± 3.6, compared to 9.91 ± 3.14 in the intervention group (*p* = 0.04). SUV_peak_ was reduced with 13.45%. All other SUV values did not significantly differ, as shown in Table 2.

**Table 2:** Radiotracer activity in salivary glands of the intervention group (bilateral + unilateral icepacks-group) versus control group (no icepacks)

Radiotracer activity	Intervention group (bilateral + unilateral icepack group; *N* = 44)	Control group (no icepacks; *N* = 45)	Absolute difference (%)	*p* value*
Right parotid gland				
SUV_max_ (mean, ± SD)	12.04 (± 3.59)	13.14 (± 4.23)	1.10 (8.37)	0.19
SUV_peak_ (mean, ± SD)	10.67 (± 3.20)	11.53 (± 3.67)	0.86 (7.46)	0.24
SUV_mean_ (mean, ± SD)	4.52 (± 1.39)	4.89 (± 1.59)	0.37 (7.57)	0.25
Left parotid gland				
SUV_max_ (mean, ± SD)	11.07 (± 3.53)	12.95 (± 4.16)	1.88 (14.52)	*0.02*
SUV_peak_ (mean, ± SD)	9.91 (± 3.14)	11.45 (± 3.61)	1.54 (13.45)	*0.04*
SUV_mean_ (mean, ± SD)	4.39 (± 1.68)	4.94 (± 1.61)	0.55 (11.13)	0.12
Right submandibular gland				
SUV_max_ (mean, ± SD)	12.83 (± 3.41)	12.54 (± 3.36)	+ 0.29 (2.31)	0.69
SUV_peak_ (mean, ± SD)	10.76 (± 2.76)	10.89 (± 3.11)	0.15 (1.38)	0.84
SUV_mean_ (mean, ± SD)	4.84 (± 1.88)	4.48 (± 1.34)	+ 0.36 (8.04)	0.31
Left submandibular gland				
SUV_max_ (mean, ± SD)	12.34 (± 3.05)	12.27 (± 3.64)	+ 0.07 (0.57)	0.93
SUV_peak_ (mean, ± SD)	10.40 (± 2.66)	10.84 (± 2.98)	0.44 (4.06)	0.46
SUV_mean_ (mean, ± SD)	4.69 (± 1.74)	4.51 (± 1.32)	+ 0.18 (3.99)	0.59

*p < 0.05 is considered significant

### Radiotracer activity in the bilateral icepack group versus control group

Patients with bilateral icepacks showed no significant differences in PSMA uptake, when compared to the control group (all *p* > 0.05): right parotid gland (SUV_max_ 11.26 ± 3.33 versus 13.14 ± 4.23), left parotid gland (SUV_max_ 11.01 ± 3.40 versus 12.96 ± 4.16), right submandibular gland (SUV_max_ 12.36 ± 3.38 versus 12.54 ± 3.36), and left submandibular gland (SUV_max_ 11.74 ± 2.78 versus 12.27 ± 3.64). SUV_peak_ and SUV_mean_ of bilateral parotid and submandibular glands in both groups did not significantly differ either. Results are summarized in Table 3.

**Table 3:** Radiotracer activity in salivary glands of the bilateral icepack group versus the control group

Radiotracer activity	Bilateral icepacks (*N* = 20)	No icepacks (*N* = 45)	Absolute difference (%)	*p* value*
Right parotid gland				
SUV_max_ (mean, ± SD)	11.26 (± 3.33)	13.14 (± 4.23)	1.88 (14.31)	0.08
SUV_peak_ (mean, ± SD)	9.98 (± 3.08)	11.53 (± 3.67)	1.55 (13.44)	0.10
SUV_mean_ (mean, ± SD)	4.22 (± 1.32)	4.89 (± 1.59)	0.67 (13.70)	0.10
Left parotid gland				
SUV_max_ (mean, ± SD)	11.01 (± 3.40)	12.96 (± 4.16)	1.95 (15.05)	0.07
SUV_peak_ (mean, ± SD)	9.89 (± 2.99)	11.45 (± 3.61)	1.56 (13.62)	0.10
SUV_mean_ (mean, ± SD)	4.44 (± 1.64)	4.94 (± 1.61)	0.50 (10.12)	0.25
Right submandibular gland				
SUV_max_ (mean, ± SD)	12.36 (± 3.38)	12.54 (± 3.36)	0.18 (1.44)	0.84
SUV_peak_ (mean, ± SD)	10.33 (± 2.60)	10.89 (± 3.11)	0.56 (5.14)	0.49
SUV_mean_ (mean, ± SD)	4.78 (± 1.70)	4.48 (± 1.34)	+ 0.30 (6.70)	0.45
Left submandibular gland				
SUV_max_ (mean, ± SD)	11.74 (± 2.78)	12.27 (± 3.64)	0.53 (4.32)	0.57
SUV_peak_ (mean, ± SD)	9.85 (± 2.30)	10.84 (± 2.98)	0.99 (9.13)	0.19
SUV_mean_ (mean, ± SD)	4.63 (± 1.74)	4.51 (± 1.32)	+ 0.12 (2.66)	0.76

*p < 0.05 is considered significant

### Radiotracer activity in the unilateral icepack group (intra-patient analysis)

In the unilateral icepack group (with cooled left side), some significant differences in SUV_max_ and SUV_peak_ were found between the left and right parotid gland (SUV_max_ cooled left side 11.12 ± 3.71 versus SUV_max_ non-cooled right side 12.69 ± 3.75, *p* = 0.00; SUV_peak_ cooled left side 9.93 ± 3.32 versus SUV_peak_ non-cooled right side 11.25 ± 3.25, *p* = 0.00). Fractional differences were 12.37% (SUV_max_) and 11.73% (SUV_peak_).

Mean SUV_mean_ between the bilateral parotid glands and all parameters on PSMA uptake in the right versus the left submandibular glands did not significantly differ. SUV_mean_ in the parotid glands decreased with 8.79%. Fractional difference of SUV values in the submandibular gland ranged from 2.43 to 3.27%. Results are shown in Table 4.

**Table 4:** Radiotracer activity in salivary glands of unilateral icepack group: (cooled) left side versus (non-cooled) right side

Radiotracer activity in unilateral (left-sided) icepack group (*N* = 24)				Absolute difference (%)	*p* value*
Left parotid gland		Right parotid gland			
SUV_max_ (mean, ± SD)	11.12 (± 3.71)	SUV_max_ (mean, ± SD)	12.69 (± 3.75)	1.57 (12.37)	0.00
SUV_peak_ (mean, ± SD)	9.93 (± 3.32)	UV_peak_ (mean, ± SD)	11.25 (± 3.25)	1.32 (11.73)	0.00
SUV_mean_ (mean, ± SD)	4.36 (± 1.75)	SUV_mean_ (mean, ± SD)	4.78 (± 1.43)	0.42% (8.79)	0.14
Left submandibular gland		Right submandibular gland			
SUV_max_ (mean, ± SD)	12.83 (± 3.23)	SUV_max_ (mean, ± SD)	13.21 (± 3.46)	0.38 (2.88)	0.15
SUV_peak_ (mean, ± SD)	10.85 (± 2.90)	SUV_peak_ (mean, ± SD)	11.12 (± 2.90)	0.27 (2.43)	0.19
SUV_mean_ (mean, ± SD)	4.73 (± 1.78)	SUV_mean_ (mean, ± SD)	4.89 (± 2.04)	0.16 (3.27)	0.22

*p < 0.05 is considered significant

## Discussion

The present study shows no significant differences in PSMA uptake comparing the patient group that was scanned with bilateral icepacks to the patient group that was scanned without icepacks. When comparing radiotracer uptake in the intervention group with the control group, however, significant differences were found with regard to radiotracer uptake in the left parotid gland. These findings were confirmed by the intra-patient analysis. Based on these results, external cooling of the salivary glands seems to have some impact. Clinical relevance of these findings, however, remains debatable.

The rationale of skin cooling in the attempt to induce vasoconstriction of the peripheral blood vessels was earlier described in the literature on chemotherapy-induced alopecia [9]. Cooling is assumed to reduce skin perfusion, decrease concentration of chemotherapy in the scalp, and consequently diminish cellular uptake by the hair follicles (due to a decreased metabolic activity of the cells). In a recently published review of 10 studies comprising 654 patients, it was concluded that scalp hypothermia indeed represents an effective preventative measure [10]. The same underlying mechanism is assumed to prevent the salivary glands for toxicity in patients undergoing ^177^Lu-PSMA therapy, hopefully reducing both specific as unspecific binding [11]. To our knowledge however, the effect of external cooling on PSMA uptake in the salivary glands was not investigated.

This is the first study that aims to systematically determine impact of external cooling on PSMA uptake in salivary glands in patients referred for ^68^Ga-PSMA PET/CT imaging in the setting of prostate cancer (re)-staging. However, several conference abstracts did address this issue earlier [12, 13]. Gaertner et al. measured PSMA uptake (SUV_mean_ and SUV_max_) in the bilateral parotid and submandibular glands of an intervention group of 25 patients [12]. In this group, bilateral salivary glands were cooled with icepacks. PSMA uptake in the salivary glands was compared to SUV values of a control group that consisted of 33 patients. As a result of external cooling, a 12 and 15% reduction of SUV_mean_ in the parotid glands and submandibular glands was found, respectively (all *p* < 0.01). Mean SUV_max_ decreased significantly in both parotid glands and submandibular glands as well (all *p* < 0.01). Bohn et al. included 50 patients undergoing diagnostic ^68^Ga-PSMA PET/CT for prostate cancer. PSMA uptake in an intervention group of 25 patients, of whom the left parotid gland was cooled with icepacks, was compared with a control group of 25 patients that were scanned without icepacks [13]. Intra-patient analysis revealed the cooled left parotid gland showing 12% less radiotracer uptake compared to the non-cooled side (range 0–42%; *p* = 0.01), a statistical significant difference that was, as expected, not found under “normal” (non-cooled) circumstances.

Several German studies assessed efficacy and toxicity of ^177^Lu-PSMA therapy in metastatic castration-resistant prostate cancer patients [14–20]. The included patients in the studies by Ahmadzadehfar et al., Heck et al., and Rahbar et al. all underwent external cooling of the salivary glands [14, 15, 17, 19]. These patients received bilateral icepacks covering the cheeks from 30 min prior to treatment, up to 4 h post-administration of ^177^Lu-PSMA. Xerostomia was reported in 4–37% of the patients, probably caused by the high uptake of PSMA ligands in the salivary glands.

In the aforementioned study by Ahmadzadehfar et al., patients received icepacks covering the parotid and submandibular glands from 30 min prior to and up to 4 h after administration of a single cycle ^177^Lu-PSMA [14]. All patients in this study underwent dynamic salivary gland scintigraphy with Technetium-99m (^99m^Tc)-pertechnetate combined with salivary gland stimulation by lemon juice 20 min post-injection on the treatment day and 8 weeks after ^177^Lu-PSMA therapy to investigate salivary gland function. Comparison of baseline with follow-up salivary gland scintigraphy did not show therapy-induced functional impairment (i.e., no change in the uptake and clearance of ^99m^Tc-pertechnetate) of the salivary glands. It was concluded that it is unclear whether cooling of the salivary glands is effective to prevent the salivary glands from therapy-related damage. Furthermore, the need for a study addressing the change in uptake of ^68^Ga-PSMA with and without icepacks was underlined.

The other study groups that did not apply icepacks reported on xerostomia in 4–7% of the patients [16, 18]. Rahbar et al. investigated 56 metastatic castration-resistant prostate cancer patients that underwent ^177^Lu-PSMA-617 therapy. Whereas mean PSMA uptake in the salivary glands was found to be greater than the dose that was absorbed by the kidneys, only 2 patients (4%) experienced mild, transient, xerostomia after 3 and 4 cycles. In the study by Kratochwil et al., 30 patients underwent 1–3 cycles ^177^Lu-PSMA-617 therapy. Two out of 30 patients developed xerostomia after the third cycle, in which prescription of artificial saliva was required. Less than 10% of the patients experienced temporal xerostomia after the first and second cycles, not affecting quality of life. It must be noted that the absolute number of patients experiencing transient xerostomia after treatment was not reported. Evaluating the results of the abovementioned studies, remarkably less xerostomia was found in the patient groups that underwent therapy without cooling of the salivary glands, when compared to the patients that were supplied with icepacks during ^177^Lu-PSMA administration by other study groups. In this respect, it is important to note that the PSMA uptake in the salivary glands in the abovementioned studies was not objectified. Differences may have been caused by bias as a result of the subjective experience of therapy-related toxicity: patients undergoing ^177^Lu-PSMA therapy that were supplied with icepacks may have been more focused on xerostomia than patients that were not supplied with icepacks. Furthermore, since it concerned a retrospective study, results may have been reported less accurately.

The results of the present study are in line with those found in the abovementioned conference abstracts. Intra-patient analysis with regard to PSMA uptake in the parotid gland indeed revealed some significant findings. Comparison of radiotracer activity in the salivary glands of the entire intervention group versus the control group confirmed these findings: a significant reduction of SUV_max_ (*p* = 0.02) and SUV_peak_ (*p* = 0.04) was found in the left parotid gland. The absolute reduction was 14.52 and 13.45%, respectively.

A direct (inter-patient) comparison of the bilateral icepack group with the control group did not reveal significant differences concerning radiotracer uptake in the salivary glands. Having a closer look to these specific results, however, marginal non-significant differences in radiotracer uptake were found regarding the SUV_max_ in the bilateral parotid glands (right side *p* = 0.08; left side *p* = 0.07). Absolute SUV_max_ reduction in the right parotid gland was 14.3 and 15.1% on the left side. These findings suggest that our results would be significantly different in a larger patient sample. On the other hand, when determining the required sample size for this study, a 40% reduction of radiotracer uptake was considered clinically relevant.

The general, but not significant observed lower mean SUV values in the submandibular glands may be explained by the argument that cutaneous cooling is not equally affective for both the parotid and submandibular glands. The first explanation for this observation might be that the parotid glands are more susceptible to external cooling than submandibular glands, due to their more superficial anatomical localization. Secondly, the parotid glands may have been cooled more effectively than the submandibular glands, causing a greater difference between the cooled and the non-cooled side. Another, although less likely, potential factor may be that the parotid and submandibular glands respond differently to cooling: causing differences in vasoconstriction, blood flow, cellular metabolism, and radiotracer uptake. A vast analysis of the assumed relation between skin temperature, perfusion of the salivary glands, and radiotracer uptake in patients undergoing PRLT may offer some clarification to the assumed underlying mechanism.

The general value of external cooling of the salivary glands to prevent of xerostomia in patients undergoing ^177^Lu-PSMA therapy remains debatable. Firstly, it is suggested that external cutaneous cooling of the skin induces reactive vasodilatation, undoing the intended vasoconstrictive effect that is assumed to prevent radiotracer uptake in the salivary glands [21]. Furthermore, the presented ^68^Ga-PSMA accumulation data are meant to predict the behavior of ^177^Lu-PSMA. However, compartment modeling of radiotracer kinetics showed the presence of a relatively rapid blood clearance and a relatively slow early elimination phase of ^68^Ga-PSMA, while the effect of external cooling on these rates is not well determined [22]. In the case of a relatively slow uptake of ^177^Lu-PSMA from interstitial or intracellular space into the salivary glands, in combination with the long half-life time of ^177^Lu, the reduction of PSMA uptake in salivary glands by cooling during a relatively short period of time can be anticipated to be even less effective.

Limitations comprise the measurements executed by one unblinded investigator, potentially affecting their accuracy. However, an experienced nuclear medicine physician randomly checked measurements. Secondly, baseline temperature of the icepacks was not standardized or systematically measured prior to application, making inter-patient differences possible, leading to potential differences in the effect of cooling and subsequent radiotracer uptake. However, the icepacks were cooled in the same fridge, in which the standard temperature of the freezer unit was approximately − 18 °Celsius. Strong points of the present study include the prospective design allowing for both inter-patient and intra-patient analysis.

Diagnostics and therapy using PSMA directing radioligands are gaining popularity. The first experiences with ^177^Lu-PSMA therapy show little side effects and with favorable toxicity profiles. However, xerostomia represents an undesirable side effect that needs to be reduced. Since the clinical relevance of our results is debatable, we suggest that long-term application of icepacks during the therapeutic procedure could be considered, but most likely does not contribute to reduction of xerostomia. Extension of knowledge on the mechanisms of non-specific uptake of PSMA ligands in the salivary glands may lead to new preventive strategies while improved treatments of salivary gland dysfunction, if these can be identified, are also important. We await the results of ongoing gene therapy trials with interest. Promising strategies encompassing intraglandular injection of several compounds (e.g., botulinum toxine), but also gene- and stem cell therapy, have been suggested to prevent xerostomia and might offer a solution to in the future [11]. Prospective studies, investigating efficacy and toxicity including oncological outcomes regarding overall survival, are warranted to direct future ^177^Lu-PSMA therapy.

## Conclusions

External cooling of salivary glands using icepacks seems to reduce ^68^Ga-PSMA uptake in the parotid glands only. However, as clinical relevance of these findings is debatable, structural application of icepacks in patients undergoing ^177^Lu-PSMA therapy in advanced prostate cancer should remain optional, but most likely does not contribute to reduction of xerostomia.

## Abbreviations

^177^Lu: Lutetium-177
^68^Ga: Gallium-68
EANM: European Association of Nuclear Medicine
GMP: Good manufacturing practice
PRLT: Peptide receptor ligand therapy
PSMA: Prostate-specific membrane antigen
SUV: Standardized uptake value
VOI: Volume of interest
